# Drivers of teacher commitment to place-based education: unveiling motivational pathways through a cognitive-affective-behavioral lens

**DOI:** 10.3389/fpsyg.2026.1781707

**Published:** 2026-03-03

**Authors:** Xiaoxia Li, Wanxia Zhu

**Affiliations:** College of Teacher Education, Northwest Minzu University, Lanzhou, China

**Keywords:** cognitive-affective-behavioral, place cognition, place emotion, place identity, place-based education

## Abstract

Despite the growing emphasis on place-based education in K-12 settings, research into the motivational mechanisms that drive teachers’ implementation of these pedagogies remains scarce. This study aims to develop and validate a conceptual model of K-12 teachers’ motivation for place-based education, based on the Cognitive-Affective-Behavioral framework. Data were collected from 255 K-12 teachers across 99 schools and analyzed using Partial Least Squares Structural Equation Modeling (PLS-SEM). The results indicate that teachers’ place cognition significantly enhances their place identity (*β* = 0.753, *p* < 0.001) and place emotions (*β* = 0.446, *p* < 0.001), which in turn positively influence their willingness to implement place-based education (*β* = 0.749, *p* < 0.001). Notably, place identity alone does not have a direct significant effect on implementation willingness. These findings confirm the cognitive-affective pathway in education and enhance motivation theory by identifying key motivational factors in place-based education. The study recommends that educational policymakers incorporate local cultural content into teacher training and organize immersive experiences to strengthen teachers’ place cognition and emotional engagement.

## 1 Introduction

Place-based education (PBE), which harmonizes educational content and pedagogical approaches with local cultural, historical, and societal contexts, is widely acknowledged as a pivotal strategy for enhancing educational quality and efficacy. Extensive empirical research has consistently demonstrated that place-based education significantly enhances students’ learning motivation and academic achievements, with this positive impact primarily attributed to its inherent alignment with students’ personal experiences and cultural backgrounds (Smith, 2007; Feger, 2006). Furthermore, empirical evidence suggests that this pedagogical approach has substantially enhanced students’ cognitive development, particularly in terms of knowledge comprehension and integration, while simultaneously deepening their understanding of local contexts and strengthening their critical thinking and problem-solving competencies (Sobel, 2004; Burbules and Berk, 1999). Moreover, this approach has demonstrated significant efficacy in fostering students’ social responsibility and civic engagement, contributing to their development as active community members (Westheimer and Kahne, 2004).

The conceptual framework of place-based education has attracted substantial scholarly attention in contemporary educational research, predominantly grounded in two seminal theoretical perspectives: Lev Vygotsky’s sociocultural learning theory and the situated learning theory articulated by Jean Lave and Etienne Wenger. The sociocultural learning theory posits that knowledge construction is fundamentally a social process, intrinsically embedded within specific cultural contexts and mediated through social interactions (Vygotsky, 1978; Cobb, 1994), Complementarily, situated learning theory conceptualizes learning as an inherently social phenomenon that emerges through legitimate peripheral participation in authentic community practices and activities (Lave and Wenger, 1991).

Despite the widespread recognition of place-based education’s significance, there exists a considerable research gap concerning the underlying motivational mechanisms that drive educators to implement this instructional approach. While extant literature has primarily focused on examining teachers’ pedagogical strategies and methodological approaches in place-based education, asserting that instructional competency is crucial for enhancing educational outcomes (Gruenewald and Smith, 2014), systematic investigation into the complex motivational dynamics remains limited. Particularly noteworthy is the conspicuous absence of empirical studies that examine the intricate interrelationships among factors influencing teachers’ implementation of place-based education through theoretical modeling approaches, or that systematically investigate the operational mechanisms through which these factors interact and function (Harris and Jones, 2019; Yemini et al., 2023).

Within the K-12 educational context, where students experience crucial developmental trajectories and cognitive transformations, educators’ pedagogical approaches exert substantial influence on students’ learning dispositions and academic outcomes (Eccles and Roeser, 2011). Therefore, the systematic development and rigorous empirical validation of a comprehensive motivational model for place-based education, specifically tailored for elementary and secondary school educators, serves multiple critical purposes: it not only addresses significant gaps in the current research literature but also substantially contributes to the advancement of theoretical frameworks and methodological paradigms that underpin evidence-based educational practices.

## 2 Theoretical framework and hypotheses

### 2.1 Theoretical framework and core constructs

The Cognitive-Affective-Behavioral (C-A-B) framework, originating from cognitive behavioral therapy (Ellis, 1962; Beck, 1976), posits that cognition shapes emotion, which in turn drive behavior. This model has been extended beyond clinical settings to inform research in educational psychology, suggesting that teachers’ instructional practices and professional well-being are influenced by their cognitive appraisals and affective states (Rimm-Kaufman and Hamre, 2010; Mansfield et al., 2016). The C-A-B framework is particularly apt for investigating K-12 teachers’ motivation for place-based education (PBE) as it provides a structured lens to examine how their understanding of the local context (cognition), their psychological and emotional connection to it (affect), and their resulting teaching intentions (behavior) dynamically interact (Gonzalez, 2023). Guided by this framework, the present study operationalizes these three dimensions through four core constructs.

Place Cognition represents the cognitive dimension. It denotes an individual’s knowledge and understanding of a place, encompassing its location, history, culture, and resources (Tuan, 1974). In this study, it refers to teachers’ awareness of local assets relevant to curriculum integration.

Place Identity and Place Emotion constitute the dual facets of the affective dimension. Place Identity is conceptualized as one’s psychological identification with a place, where the place becomes integrated into their self-concept and sense of belonging (Relph, 1976; Strandberg and Styvén, 2024). Place emotion refers to the emotional bond or connection formed between an individual and a place, including feelings of attachment, pride, comfort, or nostalgia (Altman and Low, 1992). While related, identity emphasizes a cognitive-self-evaluative component (“I am part of this place”), whereas emotion centers on the felt affective experience (“I feel attached to this place”) (Knez and Eliasson, 2017; Kyle et al., 2014). This distinction allows for a nuanced analysis of affective influences.

Intention to Implement Place-Based Education (PBE) embodies the behavioral intention dimension. It captures a teacher’s willingness and planned effort to integrate local resources into pedagogy, such as designing place-based learning activities (Smith, 2002).

In summary, within the adopted C-A-B framework, Place Cognition is posited to influence Place Identity and Place Emotion, which subsequently predict the Intention to Implement PBE. The hypothesized relationships among these constructs are elaborated in the following section.

### 2.2 Variable relationships and research hypotheses

#### 2.2.1 Cognitive variable and affective variable

Guided by the Cognitive-Affective-Behavioral (C-A-B) framework, this study posits that teachers’ cognitive appraisal of their place forms the foundation for subsequent affective responses. Foundational place theory establishes that deep knowledge and understanding of a place (cognition) are prerequisites for developing emotional ties (emotion) and a sense of identity with it (Tuan, 1974; Relph, 1976). Empirical research corroborates this sequence, demonstrating that place cognition significantly fosters place identity, which in turn enhances place emotion (Stedman, 2002), and that place cognition directly serves as the basis for identity development (Proshansky et al., 1983). A synthesis of the literature further confirms that cognitive evaluations of a place directly influence both emotional attachment and identity formation (Lewicka, 2011).

The proposed link between place identity and place emotion is reinforced by Social Identity Theory (Tajfel and Turner, 1979) and Place Attachment Theory (Altman and Low, 1992). These theories suggest that a strong place identity (a form of social identity) can strengthen positive emotional bonds, such as belongingness and pride, toward that place. In the context of K-12 teaching, this implies that a teacher’s psychological identification with their local community can deepen their emotional investment in it.

Therefore, the following hypotheses are proposed:

*H1*: Teachers’ place cognition positively influences their place identity.

*H2*: Teachers’ place cognition positively influences their place emotion.

*H3*: Teachers’ place identity positively influences their place emotion.

#### 2.2.2 Cognitive variable and behavioral variable

According to the C-A-B framework, cognition is a primary driver of behavioral intention. Place theory posits that a deep understanding of the local environment (cognition) provides the necessary foundation for educators to develop contextually relevant pedagogical approaches (Relph, 1976; Tuan, 1977). This suggests that teachers’ place cognition can directly motivate place-based educational practices. Empirical research supports this direct cognitive-behavioral link. Smith (2002) demonstrated that teachers’ understanding of their local environment significantly increases the likelihood of implementing place-aligned curricula. Similarly, Powers (2004) found that teachers’ place cognition is a significant predictor of their intention to adopt place-based education strategies, with positive perceptions of the environment correlating with greater implementation willingness. Further evidence indicates that a deep understanding of the locale enhances teaching motivation and effectiveness in place-oriented instruction (Somerville, 2010). Finally, recent studies using advanced analytical methods like PLS-SEM corroborate that an individual’s activities, experiences, and cognition of a place are significant factors in forming behavioral intentions, while also highlighting the potential mediating role of affective factors like place attachment (Dalavong et al., 2024). These findings collectively underscore the pivotal role of place cognition in shaping educators’ behavioral intentions toward place-based pedagogy. Therefore, we hypothesize:

*H4*: Teachers’ place cognition positively influences their intention to engage in Place-Based Education.

#### 2.2.3 Affective variable and behavioral variable

The Cognitive-Affective-Behavioral (C-A-B) framework posits that affective responses are critical drivers of behavioral intentions. This section argues that teachers’ emotional attachment to (place emotion) and psychological identification with (place identity) their locale directly motivates their intention to implement Place-Based Education (PBE).

The theoretical foundations for this link are robust. Emotional Geography posits that feelings toward a place fundamentally shape an individual’s behaviors and commitments within it (Davidson et al., 2005). In an educational context, this suggests that teachers’ positive place emotions, such as belonging or care, motivate them to integrate local content and experiences into their pedagogy to enhance relevance. Complementarily, Social Identity Theory explains how individuals derive part of their self-concept from group affiliations (Tajfel and Turner, 2004). Thus, a teacher’s strong identification with their school and community (place identity) reinforces their sense of professional responsibility and can strengthen their dedication to pedagogies, like PBE, that serve that community.

Empirical evidence consistently supports these theoretical pathways. Early research established that teachers with strong place identities show a greater inclination to employ PBE methodologies, integrating subject matter with students’ local environments (Lim and Barton, 2006). Similarly, positive teacher attitudes toward place emotions have been shown to enhance the implementation of environmental education practices (Zachariou et al., 2017), and teachers’ personal backgrounds and place-related beliefs profoundly shape their curricular design (Zeyer and Kelsey, 2013). Recent studies significantly deepen this understanding. Large-scale surveys confirm that teachers’ sense of place, encompassing both identity and emotional attachment, is a significant positive predictor of their professional attitudes and commitment (Zhao et al., 2020). Similarly, studies have shown that teachers’ place emotional attachment may be the main catalyst for participating in Place-Based Education (Lei and Wang, 2023).

Based on these insights, the following hypotheses were proposed for this study:

*H5*: Teachers’ place identity positively influences their intention to engage in Place-Based Education.

*H6*: Teachers’ place emotions positively influence their intention to engage in Place-Based Education.

### 2.3 Conceptual model

**Figure 1:**
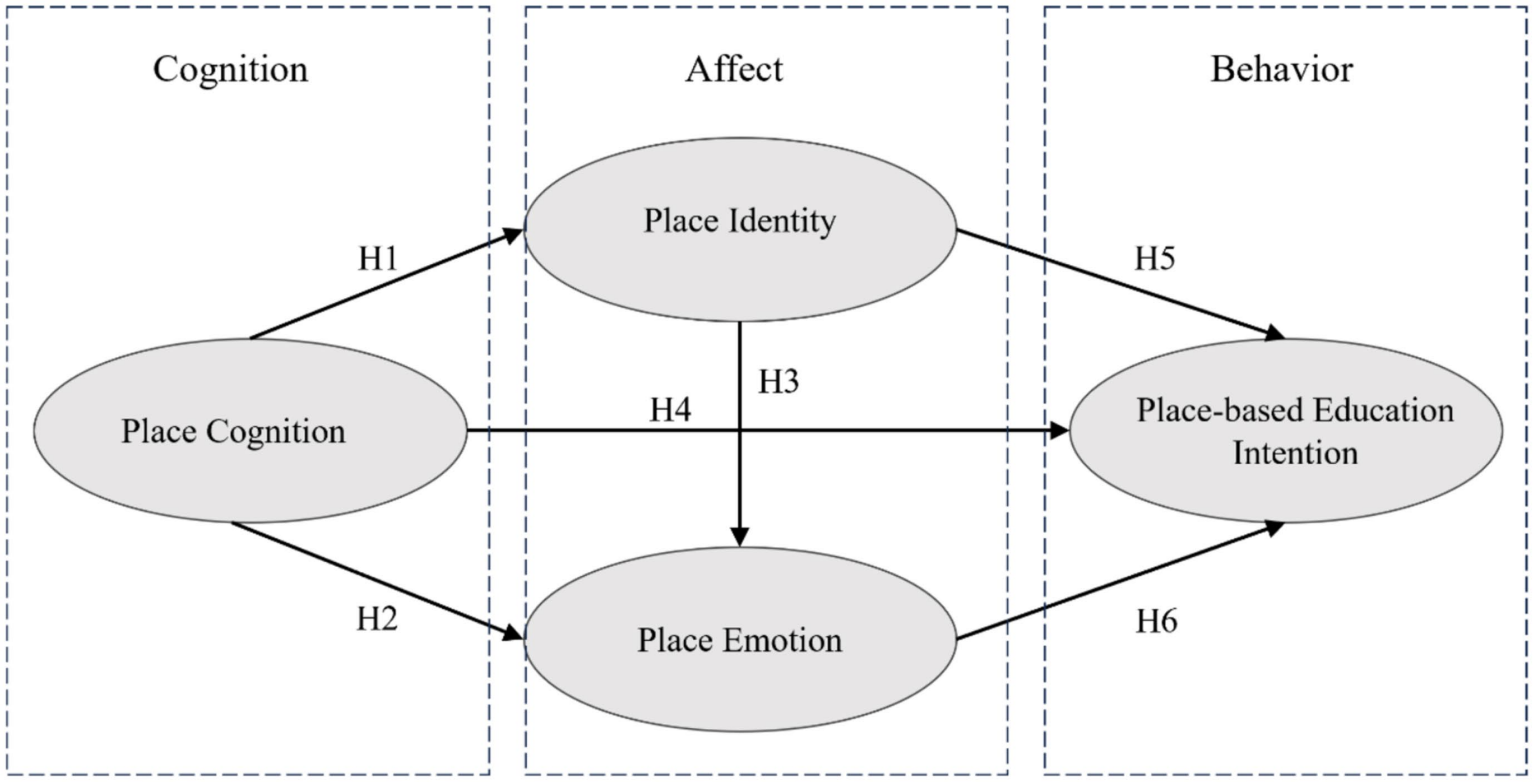
Concept model.

This study developed its conceptual model based on the Cognitive-Affective-Behavioral framework, integrating pertinent theoretical perspectives and empirical research findings. Within this model, the cognitive dimension is represented by place cognition, the affective dimension encompasses place identity and place emotion, and the behavioral dimension is defined by K-12 teachers’ intention to engage in Place-Based Education (PBE). Figure 1 illustrates the interrelationships among these variables and delineates the research hypotheses embedded within this conceptual framework.

## 3 Methodology

### 3.1 Sample

The effective sample for this study consists of 255 subject teachers from 99 K-12 schools across Northwest China. The schools are distributed across both rural and urban settings, with primary school teachers predominantly hailing from rural areas and middle to high school teachers primarily from urban locales. A stratified cluster sampling method was employed. First, the region was stratified into urban and rural districts. Then, schools were randomly selected from each stratum, including 42 primary schools, 34 middle schools, and 23 high schools (including vocational high schools). Within each selected school, subject teachers were invited to participate voluntarily. This approach aimed to ensure representation across school levels and locations. The gender distribution is nearly balanced, comprising 127 male teachers (49.80%) and 128 female teachers (50.20%). Participants’ ages range from 23 to 60 years, with a median age of approximately 40 years. In terms of teaching experience, 137 teachers (53.73%) have over 15 years of experience, while novice teachers account for 5.49% of the sample, totaling 14 individuals. Excluding one intern, all participants are tenured subject teachers. The fields of specialization among these teachers include 49 in Chinese, 37 in Mathematics, 24 in English, and 19 in Computer Science or related disciplines.

### 3.2 Instruments

The primary instrument was a self-report questionnaire comprising demographic items and scale-based questions. To mitigate potential common method bias, procedural remedies were applied during data collection, including ensuring respondent anonymity and separating scale items in the questionnaire layout (Podsakoff et al., 2003). The scales assessed teachers’ place cognition, place identity, place emotion and intention to implement Place-Based Education. All scales used a 7-point Likert scale (1 = strongly disagree, 7 = strongly agree).

#### 3.2.1 Place cognition scale

Adapted from Scott and Bruce (1994), the Place Cognition Scale includes three items that evaluate teachers’ awareness of place-based cultural, historical, and artistic resources. These items were modified to align with the Chinese cultural context and underwent a rigorous back-translation process to ensure linguistic and conceptual accuracy.

#### 3.2.2 Place identity scale

The Place Identity Scale, revised from Ramkissoon et al. (2013), consists of three items that assess teachers’ identification with the place culture and environment. These items were carefully adapted to reflect the Chinese cultural milieu and validated through a back-translation process to maintain translation integrity.

#### 3.2.3 Place emotion scale

Similarly revised from Ramkissoon et al. (2013), the Place Emotion Scale comprises three items that measure teachers’ affection for and attachment to the place area. These items were specifically adapted for the Chinese cultural context and subjected to a back-translation process to ensure their appropriateness.

#### 3.2.4 Intention to implement place-based education scale

This scale, adapted from Fornara et al. (2016), assesses teachers’ intention to implement Place-Based Education. It includes three items modified from the original focus on “place environmental conservation” to “education grounded in place cultural, historical, and artistic resources.” These modifications were tailored to the Chinese cultural context, and the translation’s accuracy was ensured through a back-translation process.

### 3.3 Data analysis methods

Quantitative data collected through the survey were analyzed using Partial Least Squares Structural Equation Modeling (PLS-SEM). The selection of PLS-SEM was based on three primary considerations: Firstly, PLS-SEM is exceptionally well-suited for smaller sample sizes. With a sample size of 255, PLS-SEM provides robust outcomes, particularly advantageous in exploratory research and complex model configurations (Hair et al., 2011). Secondly, PLS-SEM excels in managing sophisticated theoretical models that encompass multiple latent variables and pathways. The model developed for this study, grounded in the Cognitive-Affective-Behavioral framework, incorporates numerous dimensions and interactions, making PLS-SEM an effective method for estimating intricate relationships (Henseler et al., 2009). Thirdly, beyond empirical testing, PLS-SEM promotes theoretical advancement by allowing researchers to refine and enhance the model throughout the development process. This is particularly valuable for the formulation of novel theoretical frameworks (Chin, 2010). These factors collectively support the use of PLS-SEM as the analytical method for this investigation, enabling a comprehensive examination of the relationships within the conceptual model.

## 4 Results

This study utilized quantitative data collected through surveys and analyzed using the PLS-SEM approach to validate the proposed conceptual model.

### 4.1 Results of the measurement model

The measurement model was assessed based on four criteria: internal consistency reliability, item reliability, convergent validity, and discriminant validity.

#### 4.1.1 Internal consistency reliability

Internal consistency was assessed by calculating Cronbach’s alpha and Composite Reliability (CR) for all variables. According to Cronbach (1951) and Nunnally and Bernstein (1994), Cronbach’s alpha should exceed 0.800 or 0.900 in confirmatory research and 0.700 in exploratory research. Similarly, Werts et al. (1974) and Nunnally and Bernstein (1994) suggested that Composite Reliability serves as a robust alternative to Cronbach’s alpha, where CR should be greater than 0.800 or 0.900 in confirmatory research and above 0.700 in exploratory settings. As presented in Table 1, all dimensions demonstrated Cronbach’s alpha values exceeding 0.800 and Composite Reliability values surpassing 0.900. Therefore, all latent variables in this exploratory study met the recommended thresholds, indicating strong internal consistency reliability.

**Table 1:** Measurement model results for the teacher’s place-based education motivation model.

Constructs/items	Mean, STDEV, T values, *p* values of outer loadings				Construct reliability and validity		
	Factor loadings	Standard deviation	T statistics	*P* values	Cronbach’s alpha	Composite reliability	AVE
PBEI					0.975	0.984	0.952
PBEI1	0.977	0.007	130.968	***			
PBEI2	0.983	0.005	206.664	***			
PBEI3	0.967	0.010	101.530	***			
PLEM					0.921	0.950	0.864
PLEM1	0.902	0.032	28.053	***			
PLEM2	0.939	0.016	57.990	***			
PLEM3	0.947	0.013	70.847	***			
PLID					0.926	0.953	0.871
PLID1	0.935	0.022	43.013	***			
PLID2	0.953	0.013	75.111	***			
PLID3	0.911	0.034	26.576	***			
PLCO					0.938	0.960	0.890
PLCO1	0.967	0.007	137.322	***			
PLCO2	0.957	0.010	95.820	***			
PLCO3	0.904	0.035	25.975	***			

(1) PBEI stands for Place-based Education Intention, PLEM for Place Emotion, PLID for Place Identity, and PLCO for Place Cognition, the same hereinafter; (2) The significance test method for the subject factor is bootstrapping, K = 5,000; (3) ***, *p* < 0.001, the same hereinafter.

#### 4.1.2 Item reliability

Item reliability was evaluated using the PLS-SEM approach. Typically, an item’s factor loading indicates the extent to which its variance is explained by the corresponding latent variable, thereby reflecting the item’s reliability. Following Chin (1998), each item’s factor loading should be statistically significant at the 0.05 confidence level and exceed 0.700, although marginally lower values may be acceptable in exploratory research designs. As shown in Table 1, all items in the study model exhibited factor loadings greater than 0.700 and were significant at the 0.05 confidence level, demonstrating high item reliability.

#### 4.1.3 Convergent validity

Convergent validity was established by calculating the Average Variance Extracted (AVE). The AVE was derived by analyzing the variance contributions from the factor loadings of the items and the measurement errors associated with the latent variables. According to Fornell and Larcker (1981), an AVE value should be 0.500 or higher to confirm the convergent validity of latent variables. As depicted in Table 1, all latent variables in this study achieved AVE values exceeding 0.500, indicating satisfactory convergent validity.

#### 4.1.4 Discriminant validity

Discriminant validity was assessed using two methods: the Fornell-Larcker criterion and the cross-loading approach. According to the Fornell-Larcker criterion (1981), the AVE of a latent variable should be greater than the highest Pearson correlation coefficient between that latent variable and any other latent variable. As shown in Table 2, the AVE values for all latent variables exceeded their highest correlations with other variables, confirming that the model met the Fornell-Larcker criterion and exhibited strong discriminant validity.

**Table 2:** Results for discriminant validity using the Fornell–Larcker criterion.

Constructs	PBEI	PLEM	PLID	PLCO
PBEI	**0.976**			
PLEM	0.826	**0.930**		
PLID	0.725	0.843	**0.933**	
PLCO	0.917	0.828	0.753	**0.943**

(1) The values presented in the lower left section of the table denote the Pearson correlation coefficients between the constructs of the model and other constructs; (2) The values in bold represent the square roots of the Average Variance Extracted (AVE) for the respective latent variables.

The discriminant validity was further confirmed using the cross-loadings method. This approach requires that the factor loadings of each item should be significantly higher on their designated constructs than on any alternative constructs. In accordance with the guidelines outlined by Chin (1998), the factor loadings for each construct in this study surpassed those associated with any other constructs, as shown in Table 3. This finding further strengthens the model’s discriminant validity.

**Table 3:** Results for discriminant validity using cross-loadings.

Items	PBEI	PLEM	PLID	PLCO
PBEI1	**0.977**	0.816	0.712	0.895
PBEI2	**0.983**	0.805	0.709	0.899
PBEI3	**0.967**	0.798	0.702	0.891
PLEM1	0.763	**0.902**	0.901	0.755
PLEM2	0.760	**0.939**	0.705	0.759
PLEM3	0.780	**0.947**	0.736	0.792
PLID1	0.654	0.778	**0.935**	0.675
PLID2	0.718	0.805	**0.953**	0.728
PLID3	0.658	0.775	**0.911**	0.703
PLCO1	0.907	0.807	0.737	**0.967**
PLCO2	0.900	0.798	0.727	**0.957**
PLCO3	0.784	0.735	0.664	**0.904**

The bold values represent the factor loadings of each measurement item on its corresponding latent variable.

### 4.2 Structural model results

This study conducted a thorough analysis of the structural model from three key perspectives: the coefficient of determination (*R*^2^), testing of the research hypotheses, and model fit results.

#### 4.2.1 *R^2^* analysis

**Figure 2:**
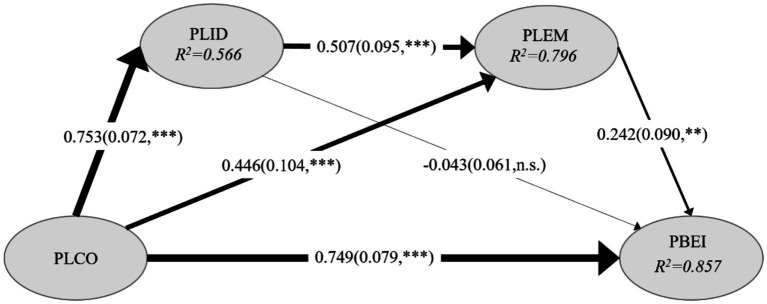
Path coefficients and their significance results of the model.

Following the confirmation of the reliability and validity of the measurement model, the next step was to evaluate the coefficient of determination (*R*^2^) for the structural model. The *R*^2^ statistic quantifies the proportion of variance in the endogenous for, serving as an indicator of the model’s explanatory power. According to Chin (1998) classification, *R*^2^ values greater than 0.670 are considered high, values around 0.333 are classified as moderate, and those near 0.190 are regarded as low. In this study, the *R*^2^ values for the endogenous latent variables were 0.566, 0.796, and 0.857, reflecting moderate to high explanatory power (see Figure 2). These results suggest that the model effectively elucidates the key variables under investigation.

#### 4.2.2 Testing of research hypotheses

In this study, we employed the PLS-SEM to evaluate the research hypotheses. The path coefficients and their significance within the structural model are presented in Table 4 and Figure 2.

**Table 4:** Hypothesis testing results.

Hypothesis	Path	Path coefficients	SD	T statistics	*P* values	Result
H1	PLCO → PLID	0.753	0.072	10.455	0.000	Supported
H2	PLCO → PLEM	0.446	0.104	4.279	0.000	Supported
H3	PLID → PLEM	0.507	0.095	5.339	0.000	Supported
H4	PLCO → PBEI	0.749	0.079	9.466	0.000	Supported
H5	PLID → PBEI	−0.043	0.061	0.699	0.484	No supported
H6	PLEM → PBEI	0.242	0.090	2.700	0.007	Supported

The specific results are as follows: The path coefficient from “Teachers’ Place Cognition(PLCO)” to “Teachers’ Place Identity(PLID)” is 0.753, with a *p*-value < 0.001, supporting Research Hypothesis 1. The path coefficient from “Place Cognition(PLCO)” to “Teachers’ Place Emotion(PLEM)” is 0.446, with a p-value < 0.001, supporting Research Hypothesis 2. The path coefficient from “Teachers’ Place Identity(PLID)” to “Teachers’ Place Emotion(PLEM)” is 0.507, with a p-value < 0.001, supporting Research Hypothesis 3.he path coefficient from “Place Cognition(PLCO)” to “Teachers’ Place-Based Education Intention(PBEI)” is 0.749, with a *p*-value < 0.001, supporting Research Hypothesis 4. The path coefficient from “Teachers’ Place Identity(PLID)” to “Teachers’ Place-Based Education Intention(PBEI)” is −0.043, with a p-value > 0.050, indicating that Research Hypothesis 5 is not supported. The path coefficient from “Teachers’ Place Emotion(PLEM)” to “Teachers’ Place-Based Education Intention(PBEI)” is 0.242, with a p-value < 0.010, supporting Research Hypothesis 6. These results suggest that, with the exception of Hypothesis 5, all other hypotheses were supported by the data.

#### 4.2.3 Model fit results

Typically, PLS-SEM does not rely on traditional global fit indices. However, Tenenhaus et al. (2004) introduced a composite fit index (GoF) that is suitable for assessing the overall fit of PLS-SEM models. Following the methodology proposed by Tenenhaus et al. (2004), the GoF value obtained in this study was 0.813. According to established standards, a GoF value greater than 0.36 indicates a high level of fit, while values around 0.25 signify a moderate fit, and values below 0.10 reflect a poor fit. Therefore, the model in this study demonstrates a high level of fit, suggesting a strong alignment between the model and the empirical data.

## 5 Discussion

### 5.1 Theoretical contributions

This study makes several key theoretical contributions. First, it successfully adapts and validates the C-A-B framework within the context of teacher motivation for place-based education, demonstrating its utility in explaining how teachers’ internal processes (cognition → affect → behavior) drive pedagogical innovation. This extends the application of the C-A-B model beyond social psychology into the specific domain of place-based pedagogy.

Second, the findings illuminate the cognitive-affective pathway in PBE motivation. The strong influence of place cognition on both identity and emotion underscores the foundational role of knowledge and understanding in fostering emotional connections. This aligns with and extends Place Attachment Theory (Altman and Low, 1992) into the professional domain of teaching.

Most notably, the non-significant direct effect of place identity on behavioral intention (H5) is a theoretically meaningful finding. This contrasts with some previous studies (e.g., Lim and Barton, 2006) and suggests that in certain contexts—particularly within the studied Chinese K-12 educational setting—a teacher’s sense of place identity may not directly translate into the intention to implement PBE. Several contextual interpretations are plausible: (1) Professional Role vs. Personal Identity: In highly structured education systems, teachers’ pedagogical decisions may be more strongly driven by professional obligations, curriculum requirements, or perceived usefulness, rather than personal place identity (Lojdová et al., 2021). (2) Emotional Mediation: Place identity’s influence may be fully mediated by place emotion, as suggested by the strong H3 path. Identity might need to be “heated” by emotional attachment to become motivationally potent for behavioral intention (Hosany et al., 2017; Knez et al., 2018). (3) Cultural Moderation: The concept of “place identity” and its behavioral consequences might be culturally construed. In collectivist settings, identity might be more tied to community expectations than to personal pedagogical choice (Zhang et al., 2021; Yang et al., 2024). This finding challenges the assumed direct affective-behavioral link and calls for more nuanced models that consider cultural, institutional, and emotional mediating variables.

### 5.2 Practical contributions

The findings yield actionable implications for key stakeholders involved in promoting place-based education (PBE).

For teacher educators and professional development designers, it is essential to prioritize the enhancement of place cognition through training that immerses teachers in the local history, culture, and ecology (Basabe et al., 2025; Gonzalez, 2023). Furthermore, fostering place emotion should be approached by organizing meaningful, positive experiences within the community—such as field trips and collaborative projects—rather than relying solely on narrative-driven identity building. Training modules should then explicitly link these developed forms of local knowledge and emotional connection to practical strategies for PBE curriculum design, thereby enabling teachers to translate understanding into pedagogical action.

School administrators play a crucial role in creating an institutional culture that values and actively supports local engagement. This involves providing teachers with the time and structural support to connect with their communities, as well as facilitating partnerships with local museums, cultural centers, and environmental organizations to supply resources and immersive experiences (Jaikrasen and Ketsing, 2025). Administrators can further legitimize the significant cognitive and emotional effort required for PBE by implementing recognition and incentive mechanisms that formally reward successful place-based education practices.

For policymakers, strategic support is needed to create an enabling ecosystem for PBE. This includes funding the development and dissemination of locally relevant instructional materials to reduce the preparatory burden on teachers (Kassim et al., 2025). Additionally, integrating place-consciousness and community engagement into official teacher standards and evaluation frameworks would signal its importance as a professional competency. Finally, allocating dedicated funding to sustain school-community partnership initiatives is vital for effectively bridging the gap between educational institutions and their local contexts.

## 6 Limitations and future research directions

### 6.1 Study limitations

Several limitations of this study should be acknowledged. First, the cross-sectional design and reliance on self-report measures limit the ability to infer causality and introduce the potential for common method bias. Second, the exclusive focus on schools in Northwest China constrains the cultural generalizability of the findings to other regions or educational systems. Third, the study assessed teachers’ behavioral intentions rather than their actual classroom implementation of PBE, leaving a gap between reported attitudes and observable practice. These limitations provide a clear rationale for the future research directions outlined below.

### 6.2 Future research directions

Building upon the identified limitations, future research should pursue three integrated directions to advance the theoretical and practical understanding of place-based education (PBE).

First, to address the constraints of cultural specificity and limited generalizability, cross-cultural comparative studies are essential. Future work should examine how the expressions and interrelationships of place cognition, identity, and emotion vary across diverse regional and national educational contexts. Such comparisons will help disentangle universal psychological mechanisms from culturally contingent factors, directly informing the development of more adaptable and targeted PBE policies and training frameworks (Roazzi et al., 2009).

Second, to overcome the methodological constraints of the cross-sectional design and reliance on self-reported intentions, a shift toward longitudinal and mixed-methods designs is critical. Longitudinal studies are needed to trace the causal pathways and temporal evolution of teachers’ place attachments and their translation into practice. Complementing surveys with qualitative methods (e.g., interviews, classroom observations) will provide richer, contextualized data on actual teaching behaviors and the complex process of PBE implementation, moving beyond the measurement of intentions alone.

Third, to bridge the gap between intentions and actual behavior and to elucidate the underlying processes, research must systematically investigate key mediating and moderating variables. Examining mediators such as teachers’ self-efficacy for PBE and educational beliefs, alongside contextual moderators like institutional support and community partnership quality (Chan et al., 2008), will provide a more nuanced model of how place-oriented dispositions ultimately transform into sustainable classroom practices. This line of inquiry is vital for developing effective, multi-level interventions that support teachers in their PBE journey.

## Data Availability

The raw data supporting the conclusions of this article will be made available by the authors, without undue reservation.
